# Status of Human-Made Radioactive Materials in a Biophysical Constrained World

**DOI:** 10.1021/acsomega.5c12091

**Published:** 2026-01-31

**Authors:** Fanny Böse, Friederike Frieß

**Affiliations:** † Workgroup for Economic and Infrastructure Policy, 26524 Technische Universität Berlin, Straße des 17. Juni 135, 10623 Berlin, Germany; ‡ 27270 BOKU University, Institute of Safety and Risk Sciences, Department of Landscape, Water and Infrastructure, Peter-Jordan-Straße 76, 1190 Vienna, Austria

## Abstract

The discovery of nuclear fission nearly a century ago not only caused the production of significant quantities of radioactive materials, but also purposefully brought naturally occurring radioactive substances from the Earth’s crust to the surface through mining. The use of these materials, particularly in nuclear weapons, has profoundly influenced our understanding of the Earth system, driven by the military’s need to study the effects of nuclear explosions. This introduced the alarming possibility of irreversible, severe consequences on a global scale within a very short time frame. Despite their critical significance, radioactive materials are scarcely addressed within the Planetary Boundaries framework. This oversight may stem from the challenge of defining control variables or safe operating spaces for the two most consequential release pathways: nuclear power plant accidents and nuclear explosions. Applying the precautionary principle in this context would advocate for a world free of nuclear weapons and a cautious, well-considered approach to the use of nuclear energy.

## Graphical abstract

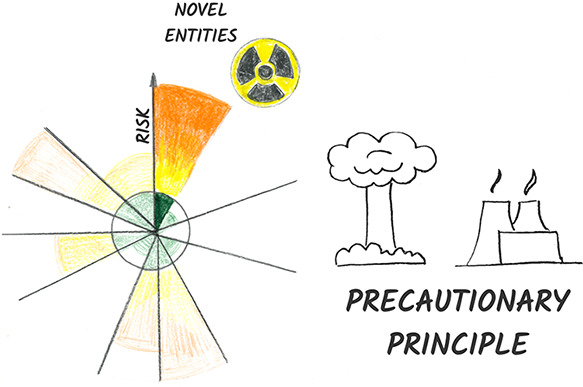

## Introduction

It is widely recognized that human influence alters the Earth’s systems on a planetary and irreversible scale. Paul Crutzen and Eugene Stoermer proposed the term Anthropocene in 2000 to describe a new part of Earth’s history that highlights human dominance over environmental systems on a global scale. The term was seen as a supplement to the Holocene. Now, it is often used to distinguish current and future living conditions from Holocene conditions (meaning the relatively stable Earth system processes in the last millennia, e.g., climate, oceans health, biodiversity). The discussions about the starting point of the new epoch deemed radioactive materials, in particular plutonium which was produced for nuclear weapons and mainly released by nuclear weapons testing in the 1950s and 1960s, as a relevant and suitable marker.

The term Anthropocene is closely linked to the global sustainability concept of the Planetary Boundaries, as this framework addresses the global impact of humans on major Earth system processes. The main purpose of the Planetary Boundary framework is to quantify the status of those processes to determine a *“safe operating space”* based on Holocene-like conditions.^1^ The safe operating space is a concept to describe the environmental limits within which humanity can continue to develop and thrive without destabilizing the planet. Critiques argue that this concept should at least be supplemented by societal boundaries to be considered *“just”*.^2^

Interestingly, the Planetary Boundaries have almost exclusively sidelined the release of human-made radioactive materials so far. Only some scholars name radioactive materials among all other (human-made) substances that are released into the environment.^1,3^

In this paper, we give an overview of the relevance of human-made radioactive materials to our understanding of the Earth as an interconnected system shaped by humans, in particular when used in and produced by nuclear explosions. This understanding is a prerequisite to understand the Planetary Boundaries concept.

We start by highlighting the importance of radioactive materials releases of the 1950s and 1960s to the Anthropocene concept. Plutonium from nuclear weapons testing was chosen as a suitable marker for the Anthropocene because it was clearly distinguishable, and it appearance coincides with other major environmental drivers such as carbon dioxide or microplastics. However, radioactive materials are more than a marker. We will show how deeply our understanding of the Earth system is intertwined with the military needs during the Cold War, especially the need to understand the effects of nuclear explosions.

We then shift the focus to the development of the Planetary Boundaries concept and the role of radioactive materials as a novel entity of global concern. Here, we take a deeper look at the different origins of radioactive materials and provide an overview of different release pathways. We will show the difficulties in quantifying a safe operating space for radioactive materials and discuss possible implications of the precautionary principle.

## Importance of Radioactive Materials for Earth System Studies

Radioactive materials were first identified at the end of the 19th century. While some radionuclides naturally exist within the earth’s crust, human activities such as mining have brought them to the surface. However, it was not until the discovery and understanding of nuclear fission in 1939—a process capable of releasing immense amounts of energy—and the subsequent realization of its potential military applications that large-scale production of radionuclides began.

In the following, we will demonstrate how the necessity of understanding the consequences of the military applications of nuclear fission advanced our comprehension of the Earth as an interconnected system. Subsequently, we will explore the selection of plutonium as a marker for the proposed, yet rejected, geological epoch known as the Anthropocene.

### Effects of Nuclear Explosions as a Motivation for Earth System Studies

The idea that nature could be controlled and manipulated in favor of warfare did not arise with the Cold War. Julius Caesar was famous for his scorched earth tactics. But those were aimed at making the land unusable after fighting. During World War I, the use of chemical weapons not only targeted the enemy’s body, but also the surrounding environment. Thus, military strategies involved weapons that affect the elements necessary for human survival. The scale of destruction via weapons changed over time, in particular with the development of nuclear weapons. Most of the releases of radioactive materials into the environment occurred due to nuclear explosions, in particular as a result of atmospheric weapons testing.

The first nuclear weapons tests were mostly conducted on ground level and were assessed for local environmental effects because the debris cloud did not penetrate the stratosphere.^4^ The fallout prediction unit of the U.S. Health and Safety Laboratory (HASL) was originally set up to predict radioactive fallout to about 200 miles (320 km) distance from ground zero. Unlike the now widely accepted linear-no-threshold model, the American Energy Commission (AEC) followed the concept of a permissible dose, implying that certain levels of radiation exposure do no harm This was another argument in favor of investigating only local and comparable strong radiation effects. However, a *“high-altitude”* (or atmospheric) shot in 1951 resulted in the detection of radioactive dust over 3000 km away, since particles were lifted high into the air and carried so far, extending the geographical scope of radioactive monitoring. The atmospheric weapons testing and the consequential widespread radioactive fallout led the military to track global fallout.^4^ The military’s motivation for analyzing and understanding the effects of nuclear explosions was driven by two national security reasons: First, knowledge of the consequences is necessary to protect one’s own military units and population from negative effects. Second, a profound understanding of the long-range effects of nuclear explosions enables the detection and estimation of the capabilities and yields of other countries’ nuclear testing. Consequently, unprecedented funding went into Earth system research. The complex modeling of the global effects of nuclear testing required high computing capability, thus also advancing supercomputing capabilities.^5^

Using those models, meteorologists showed that radioactive particles entered the upper stratosphere and mapped stratospheric wind patterns.^6^ Radioactive fallout created human-made tracers in global environmental systems, which triggered studies about global circulation. One example is the tracking of the global distribution of Sr-90 in the food chain starting as early as in 1957. This research area focused less on the military need, but on questions related to ecology and population studies. Its results led to massive public concern about the possible consequences of nuclear weapons use - and to the Limited Test Ban Treaty (LTBT), which came into force in 1963. For verification purposes, the LTBT required global monitoring systems to track radioactive elements, as well as seismic and acoustic monitoring capabilities and supercomputing. Later, satellite systems were added to this infrastructure.^4^

The nuclear weapons inventory increased strongly in the 1950s and 1960s and so did their yield.^7^ A second wave of understanding of the effects of nuclear weapons emerged in the 1980s. The improved modeling capabilities allowed for the description of the global consequences of a nuclear war, especially the fall in average temperature as a result of intense soot injection into the atmosphere: *“Nuclear war could destroy the biological support system of civilization”*.^8^ This potential threat is still present and valid: “*There is the risk of destruction of entire ecosystems and the extinction of species*, “ stated the G7 Foreign Affairs Ministers after their Meeting in April 2024. This is seconded, e.g., by *“The Nobel Laureate Assembly Declaration for the Prevention of Nuclear War”* and scientific evidence on the potentially irreversible consequences of radioactive contamination from accidents of nuclear power plants as well as from the effects following nuclear weapons explosions.^9^

The development of nuclear capabilities alongside with the so-called Single Integrated Operational Plan (SIOP) that outlined strategies, targets and procedures for deploying nuclear weapons in case of conflict shows how the global biosphere became the ultimate domain of security. The arms race mobilized technoscience to understand the planet as an global ecosystem.^10^ This led to unprecedented advances in climate and Earth system research that still shape our understanding of the planet today. Additionally, it coproduced visions of security and planetary control.

### Plutonium as a Marker for the Anthropocene

During the 2000s, radioactive materials emerged as a potential marker in discussions surrounding the Anthropocene—a proposed epoch that characterizes humanity’s profound influence on global environmental systems. Broadly, the Anthropocene signifies a period in which human activity has become a dominant force driving planetary change. While the term has faced criticism for obscuring accountability and has not been formally recognized as a geological epoch succeeding the Holocene, it has nonetheless become a widely accepted concept within scientific discourse.

The beginning of the Anthropocene was discussed and then connected to humans’ growing environmental impact since the 1950s, sometimes called the *“Great Acceleration”*. The Great Acceleration refers to the dramatic, exponential increase in human activity and its impacts on Earth that began around the mid-20th century[Fn fn1]. The Anthropocene is visible by different indicators such as increasing carbon dioxide emissions, ocean acidification, sea-level rise, rising global temperatures and biodiversity loss, which grow at exponential rates, resulting in increasing environmental damage.

To define a starting point for a new geological epoch, a so-called global boundary "*Stratoype Section and Point*” has to be identified. This physical reference point marks a clear global shift to a new geological time period and is often referred to as the *“golden spike”*. It is the agreed-upon lower boundary of a new geological epoch. Several potential markers had been under discussion to mark the beginning of the Anthropocene. In the end, the decision was made in favor of a sediment core drilled from the bottom of Crawford Lake (Ontario, Canada). This sediment captures chemical traces of the fallout from nuclear explosions and all other kinds of human-made marks, such as combustion particles and changed biotic populations.^11^ From this sediment core, the 1950 layer (*“strata”*) containing plutonium was proposed as the reference point.

There are several reasons why plutonium is a suitable choice for a golden spike. Plutonium is human-made. Before the military-industrial complex started producing plutonium for nuclear weapons, only very small amounts, dubbed *“traces"* of it appeared in nature. As a result of the atmospheric nuclear weapons testing in the 1950s and 1960s, plutonium is now globally distributed and will remain there for a long time. The most relevant plutonium isotope for nuclear weapons, plutonium-239, has a half-life of 24,000 years. In the concept of the Anthropocene, plutonium serves as a marker, which coincides with other major environmental drivers of the Great Acceleration.^12,13^

After all this preparatory work, the Anthropocene as a geological epoch following the Holocene was rejected in 2024 by the international Subcommission on Quaternary Stratigraphy (SQS).^14^ One argument against is the fact the human transformation cannot be attributed to a single starting point in time but should be rather seen as an ongoing, intensifying planetary event.^15^ Social science critiques stress that the Anthropocene functions less as a precise geological epoch and more as a contested narrative about responsibility, temporality, and power. However, it captures accelerating geophysical and biochemical changes in the Earth system, making it valuable despite its limitations.

As mentioned above, plutonium serves as a marker for the Anthropocene due to its properties such as persistence, global distribution and its coincidental release in the 1950s. Its release via the use in nuclear weapons had a significant impact on our understanding of the Earth system. But radioactive materials are not acknowledged as a major environmental concern in many frameworks. In the following, we will take a closer look at the treatment of radioactive material in the framework of the Planetary Boundaries.

## Representation of Radioactive Materials in Planetary Boundaries

In sustainability sciences, Earth‘s systems are studied with respect to the environmental impacts that are accelerating, as highlighted with the term Anthropocene. The Planetary Boundary framework aims to determine a safe operating space that aims to not cross thresholds and to maintain a safe distance from those.^1^ One of the nine Planetary Boundaries called *“novel entities”* is dedicated to human-made substances or organisms that are new to the Earth system and cause harmful, large-scale, and sometimes irreversible impacts on the environment or human health.^16^ Novel entities include new entrants that nature has never dealt with before in such quantity or form. Examples are synthetic chemicals or microplastics. The impact of these entities on the Earth system as a whole remains largely unstudied.^3^ This is especially true for radioactive materials.

### Short Historic Synopsis of Radioactive Materials in Planetary Boundaries

Böse et al.^17^ have summarized the evolution of the planetary boundary framework in regard to radioactive materials and have found that there exists a lack of analysis for radioactive materials. Even more so, they find that radioactive materials were considered in the initial proposal of the framework,^1^ but even then the focuse was on radioactive waste as an example for a novel entity. Subsequent literature then did not consider radioactive materials at all until recently. Richardson et al. (2023) provided a more comprehensive term and acknowledged radioactive materials as *“anthropogenically mobilized radioactive materials, including nuclear waste and weapons”* as a group of novel entities that should be considered.^3^

In our understanding, the term *“anthropogenically mobilized radioactive materials”* includes several groups of radioactive materials. The following classification is building upon the previous work of the authors:^17^

- Radioactive nuclides, which are created through nuclear processes during power plant operations and other facilities needed for the civil use of nuclear energy. In an ideal case, most of this material is contained in radioactive waste which can be disposed according to its properties such as heat, activity and half-lives. The amount of radioactive materials produced by nuclear power plants during building, operation, and commissioning is by far the largest of human-made radioactive materials. There exists no global tracking of global stockpiles. It is estimated to be more than 400,000 tons of spent nuclear fuel, the main component of high-level radioactive waste. Additionally, there is always a certain amount of accepted emissions of radioactive material into the biosphere during operation. In case of nuclear accidents, the release of radioactive materials can be (and has been) increased by orders of magnitude.
- Radioactive materials used in nuclear weapons and created by nuclear explosions. Nuclear weapons consist of either uranium, plutonium, or both. As of 2024, global stocks of highly enriched uranium are 1240 tons and global stockpiles of separated plutonium are 565 tons. Similar to the radioactive waste from nuclear power plants, most of this material is present in a solid form with known characteristics such as composition and amount. Once a nuclear weapon is detonated, new radioactive materials, mainly activation and fission products, emerge. Their radioactive remnants of nuclear explosions still make up a relevant proportion of the radiation exposure for the global population, they were the most significant cause of exposure to man-made environmental sources and have devastated several sites.
- Radioactive materials are also used in medicine and industry. It is estimated that more than 10,000 hospitals around the world use radioactive materials, both in sealed sources and as tracers. Most of those are very short-lived, sealed sources and thus of limited concern. They are mostly produced in research reactors which, just like power reactors, produce radioactive materials through fission and activation and carry the inherent risk of accidents. Their nuclear inventory is usually far smaller than then inventory of large-scale nuclear power plants for energy production.
- Radioactive materials that have not been created by human activities, but which have been *“surfaced”* by human activities, such as mining or drilling (compare, e.g., UNSCEAR (2000)). Those are often referred to as *“Naturally-Occuring Radioactive Materials”* (Norm). The potential environmental and health risks associated with uranium increase significantly when it is mined and processed into uranium oxide (commonly known as yellowcake). During these stages, radioactive dust, waste byproducts (known as tailings), and other residues may be released, all of which can contain hazardous radioactive substances.

These groups are based on the origin of the radioactive material rather than on specific nuclides. Several nuclides are part of more than one group. Plutonium isotopes, for example, only occur in trace amounts in nature. They are produced when uranium is irradiated in nuclear power plants. Then they are either extracted for use in nuclear weapons or discarded as radioactive waste.

The first three groups consist of radioactive materials created by human activity, while the last group consists of radioactive materials that have existed independently on Earth. Human activities have mobilized and transformed these natural materials, making them significant for interactions with the biosphere. This dimension of radioactive materials is often overlooked in public discourse. As Gabrielle Hecht hargues, uranium is only considered *“nuclear”* from a certain point in the nuclear fuel cycle.^18^

In addition to emphasizing the often “overlooked” nuclear materials, our grouping approach provides a straightforward way to understand the potential sources of radioactive materials. This clarity would not be achieved using other parameters, such as radiotoxicity, half-life, or bioaccumulation potential, which require a more in-depth understanding that is not essential for the scope of the following discussion.

### Release Pathways for Radioactive Materials

When looking at novel entities in the Planetary Boundaries, two parameters are of major interest: possible release pathways and control variables to define a safe operating space. There exists a first overview of possible release pathways and control variables in the literature.^17^ However, the pathways are not further classified or assessed, although they have very different characteristics. We grouped them according to possible quantity of release, the nature of the release (immediate vs steady and predictable) and the scale of distribution relative to one point of release (see Table 1). *“Low quantity”* in our understanding does not require immediate action, while *“predictable”* means that the release is foreseen or even expected. Other options for classification could be radiotoxicity or half-lives, both of which are radionuclide-dependent and much more difficult to assess or political variables such as accountability, compensation regimes, and governance gaps.

**Table 1:** Overview of Relevant Radioactive Material Release Pathways Grouped According to Their Origin^a^

release pathway	quantity	nature	distribution
nuclear weapons explosions	high	immediate	regional to global
nuclear accidents	variable	immediate	local to regional
routine operations	low	predictable	local to regional
leakages from storage and disposal	low	predictable	local to regional
uranium dispersion (mining)	low	predictable	local to regional

a Quantity is defined according to the fact whether immediate action is required or not. "*Nature"* differentiates between expected and unexpected releases.

The most obvious release pathway that requires immediate action is the use of nuclear weapons. The importance of these pathways is illustrated in historic weapons testing and their use. Besides the two nuclear weapons dropped on Hiroshima and Nagasaki in 1945, there were more than 2000 nuclear weapons tests. Almost 500 of them were atmospheric, meaning radioactive material was lifted high into the atmosphere and transported globally through atmospheric circulation. Radioactive contamination is inherent to use and testing of all nuclear weapons. There can be no critical explosion of nuclear weapons without producing radioactive material. The potential irreversible effects of nuclear war on the environment are shown by various studies, ranging from decline in global surface air temperature via soot injections from fires caused by nuclear explosions to the changing state of the ocean composition.

Another significant and sudden introduction of radioactive material into the biosphere occurs through nuclear accidents. Most accidents resulted in only minor releases, but two events have been classified as major accidents on the International Nuclear and Radiological Event Scale (INES): Chernobyl in 1986 and Fukushima Daiichi in 2011. Clean-up and containment efforts are ongoing at both sites. Even though the likelihood of such an event depends on technical and political aspects such as choice of reactor technology and regulatory oversight, the extent to which their effects are localized depends heavily on meteorological and geographical conditions. Nuclear accidents are unpredictable by nature. Their prevention involves interests which often lead to trade-offs, e.g., between safety and profitability. One example is the state-industry collusion in Japan, which became apparent after the Fukushima Daiichi accident.

Therefore, these two types of releases represent the most critical pathways, as they demand immediate action to mitigate harm.

Another pathway involves the planned release of radionuclides during routine operations of nuclear facilities, such as the operation of nuclear reactors and fuel reprocessing plants. These routine releases are typically subject to regulatory oversight and result in the gradual dispersion of airborne or waterborne radioactive substances over local to regional areas. Releases during planned operations are monitored and can be quantified using various metrics like activity or radiotoxicity. Facilities are required to comply with regulatory thresholds and adhere to the ALARA principle (As Low As Reasonably Achievable), where the term reasonable also incorporates economic considerations. *"Reasonable"* is therefore (also) a code for balancing safety against profitability. The pathway "*routine operations*" also includes production and use of radionuclides for medical and industrial purposes.

All civil and military applications of nuclear technology generate nuclear waste. This waste can be classified based on various parameters, such as its activity level or decay heat Ideally, this waste is appropriately stored and eventually disposed of in a manner that minimizes risks. Although the dumping of radioactive material into the sea is now prohibited, achieving absolute containment of radioactive material is not feasible. Over time, containment barriers will inevitably degrade, leading to the gradual—and potentially accelerating if a certain barrier breaks—release of radionuclides. These releases can contaminate local soils and water systems for extended periods.

Particularly concerning are above-ground storage sites, which face increasing risks due to climate change, including extreme weather events, rising sea levels, and the potential for military conflicts. The severity and extent of environmental contamination in such scenarios vary significantly depending on the specific circumstances. However, proactive planning and intervention can help strengthen containment measures to mitigate these risks to some extent.

The final pathway is one where humans do not directly produce radionuclides but instead mobilize them from their originally localized state, where they had limited interaction with the environment. Not only uranium mining and milling, but solid mineral mining in general leads to the release and distribution of radionuclides. South Australia, e.g., explicitly includes also *“operations for the processing of petroleum”* into the definition of mineral processing in its *“Radiation Protection and Control Regulations”*. Besides the release during mining and subsequent processing of the extracted material, especially uranium mining produces a relevant amount of radioactive waste. The U.S. Environmental Protection Agency recommends avoiding abandoned uranium mining sites also for possible radiation exposure. Similar to routine operations in nuclear facilities, regulations are in place that must be followed Countermeasures are implemented to minimize worker exposure and limit releases into the surrounding environment.

Overall, release pathways vary widely, ranging from low, slow, and continuous emissions to large, early (as in quick with no time for preparation) releases associated with “one-time” events such as accidents or the use of nuclear weapons. The latter pathways are characterized by significant uncertainties and unpredictability. These residual risks remain inherently tied to the use of nuclear technology.

It is important to emphasize that controlled and steady releases of radionuclides should not be dismissed as inconsequential. Even small amounts of radioactive material can accumulate in living organisms over time. Our understanding of the long-term effects of low-dose exposure is still incomplete.^19,20^ The currently applied linear-no-threshold hypothesis and the ALARA principle, which are cornerstones of nuclear regulation, reflect this uncertainty by using the precautionary principle as the underlying ethical concept.

### Precautionary Principle and a Safe Operating Space

In the case of uncertainties, the Blanetary Boundaries literature suggests to involve normative judgments according to the precautionary principle. The precautionary principle is a risk management approach used when an action, policy, or technology might cause serious or irreversible harm to human health or the environment, but where there is still scientific uncertainty about the risks. It is, e.g., detailed in Article 191 of the Treaty on the Functioning of the European Union.

Persson et al. (2022) propose that the Planetary Boundaries concept for novel entities is exceeded and argue, mainly for plastics, that the monitoring cannot keep up with the increasing volumes and the pace of releases so that the release is out of control and beyond *“global capacity for management”*.^21^ Moreover, the release should be set to zero unless the substances are certified as harmless and are being monitored.^3^ Such argumentation could also be derived from the Montreal Protocol, which has set targets for harmful substances destroying the Ozone Layer.^22^ It could be argued that even though nuclear matters involve national security, secrecy, and deterrence aspects this does not justify a totally different approach.

Nevertheless, a zero release does not seem feasible for the management of radioactive materials. Some radioactive materials are indispensable and currently irreplaceable in cancer treatment and various medical applications, where alternative diagnostic and therapeutic methods are not viable substitutes. To mitigate the release of radioactive substances and limit exposure to ionizing radiation, extensive control and regulatory measures have been established. Only minimal quantities of radioactive materials are required, and these are typically generated in research reactors. Compared to large-scale nuclear power plants, research reactors contain only a small fraction of the radioactive inventory that could potentially be released. But even those research reactors are increasingly being replaced by spallation sources to provide neutrons for research purposes, and it is possible that spallation sources may fully replace them in the future.

Regulatory measures also apply to nuclear power plants. But the very nature of those highly complex facilities results in the fact that these control and mitigation strategies can fail to function as effectively as intended. Significant efforts have been made by the nuclear industry to demonstrate, especially through the application of risk assessment methods, that the likelihood of such accidents recurring is low. However, such incidents persist. The consequences can be by far more severe compared to accidents in research reactors because of their higher radioactive inventory. This holds also true for the next generation of nuclear power plants that claim higher safety levels. The risk of a nuclear accident cannot be zero. Defining a control variable for this kind of releases seems impossible.

When evaluating the risks associated with nuclear power plants, nuclear energy is sometimes assessed as essential to mitigate climate change. This belief has led to ambitious plans to expand nuclear’s share in the global energy mix, even though these plans stand in deep contrast with the actual status of nuclear energy. This discussion is beyond the scope of the article and assessed elsewhere, see for example M.V. Ramana’s recent publications. But we want to highlight that the discussion about climate change and a safe operating system (which includes climate change) is incomplete when the consequences of radioactive material releases are not included.

The second possible release pathway involving large and early releases also offers only limited opportunities for intervention to mitigate their impacts. Besides the direct physical effects of destruction, nuclear explosions produce radioactive material, which is not contained in any form. The humanitarian consequences were made evident by the bombings of Hiroshima and Nagasaki and nuclear testing. Consequently, the development, maintenance, and modernization of nuclear weapons arsenals are often justified by the concept of nuclear deterrence.

This concept is based on the premise that the threat of an inevitable and catastrophic retaliatory strike serves to deter the initial use of nuclear weapons. Importantly, for this strategy to be effective, nuclear weapons do not need to be actively deployed in conflict. A substantial body of research exists on the theory and practice of nuclear deterrence. However, within this discourse, the environmental and humanitarian consequences of nuclear weapons were often overlooked until more recent years. From a critical social science perspective, deterrence is not merely a strategy but an ideology that justifies extensive military expenditures and reinforces global power hierarchies.^23^

But even if deterrence works: nuclear weapons are not only detonated during military conflicts; they are also tested. Even if a comprehensive treaty banning all nuclear weapons tests was in place, it would not guarantee universal adherence, as not all states may become signatories. Furthermore, such a treaty would not eliminate the risk of accidents involving nuclear weapons. Eric Schlosser has compiled an impressive account of numerous incidents, many of which resulted in near-misses where sheer luck prevented catastrophe. This affirms Charles Perrow’s assessment that accidents are not just bad luck, but systemic inevitability of complex, tightly coupled sociotechnical systems also holds true in the military realm which should inform nuclear disarmament negotiations.

## Future Outlook

Radioactive materials represent a profound human-induced change to the environment. They should be considered within the Planetary Boundaries framework. Among the various release pathways, two are of particular concern due to their potential for catastrophic consequences: nuclear accidents and nuclear explosions. Understanding and addressing these risks is essential for maintaining the stability of the Earth system.

Nuclear power plants, as highly complex systems, are inherently vulnerable to major accidents. The Fukushima Daiichi disaster serves as a stark reminder of this reality. Sociologist Charles Perrow captured this concern succinctly when he stated: *“Some complex systems with catastrophic potential are just too dangerous to exist, because they cannot be made safe, regardless of human effort”*.^24^ Despite extensive safety measures and regulations, the inherent risks of nuclear power remain, especially when considering the long-term consequences of radioactive releases. This raises important questions about the role of nuclear energy in a world where alternative low-carbon energy sources are increasingly viable.

The routine operation of nuclear power plants and research facilities also contributes to the release of radioactive materials into the atmosphere. This occurs during various stages: the front end, when fissile material is surfaced and separated; during operation; and in the back end, when safe storage of radioactive waste is required. Assessing the impact of low doses from these materials remains challenging, while the necessity of medical and research applications—irreplaceable by alternative methods—is indisputable. To establish effective control measures, a deeper understanding of potential release pathways is essential. This includes a comprehensive understanding of how radioactive materials are transported within the environment.

The military use of nuclear technology is intertwined with the civil use. It is evident that the use of nuclear weapons poses an existential threat to humanity and the environment, with even a single detonation capable of causing devastating and long-lasting consequences. While the Treaty on the Prohibition of Nuclear Weapons (TPNW) reflects growing international concern, its effectiveness is limited by the absence of nuclear-armed states as signatories.

Future research should also adapt to recent developments in the planetary boundary literature, which extended the framework to not only determine a safe space, but also a just space for humanity.^25^ A call to study a safe and just space for novel entities in particular was recently published.^2^ Future research should therefore include the justice aspects of radioactive materials in the Planetary Boundary framework. For example, the long timeframes for managing the storage of radioactive materials affect several generations. The slow progress to find deep geological repositories shifts the burden to future generations. Moreover, weapons tests and waste storage sites are predominantly affecting indigenous people.

## Footnotes

1 Critics argue that the Great Acceleration is really the acceleration of fossil capitalism and military-industrial complexes, not humanity as such. Specific states, militaries, and industries influenced our environment, not “humanity.”
